# Performance of MR fusion biopsy, systematic biopsy and combined biopsy on prostate cancer detection rate in 1229 patients stratified by PI-RADSv2 score on 3T multi-parametric MRI

**DOI:** 10.1007/s00261-024-04753-3

**Published:** 2025-01-18

**Authors:** Hannah H. Riskin-Jones, Alex G. Raman, Rushikesh Kulkarni, Corey W. Arnold, Anthony Sisk, Ely Felker, David S. Lu, Leonard S. Marks, Steven S. Raman

**Affiliations:** University of California, Los Angeles, Los Angeles, USA

**Keywords:** Prostate Cancer, Biopsy, MRI

## Abstract

**Purpose:**

We analyzed the additional value of systematic biopsy (SB) to MR-Ultrasound fusion biopsy (MRgFbx) for detection of clinically significant prostate cancer (csPCa), as increased sampling may cause increased morbidity.

**Materials and methods:**

This retrospective study cohort was comprised of 1229 biopsy sessions between July 2016 and May 2020 in men who had a Prostate Imaging-Reporting and Data System (PI-RADSv2) category ≥ 3 lesion on 3 Tesla multiparametric MRI (3TmpMRI) and subsequent combined biopsy (CB; MRgFbx and SB) for suspected prostate cancer (PCa). Cancer detection rates (CDR) were calculated for CB, MRgFbx and SB in the study cohort and sub-cohorts stratified by biopsy history and PI-RADSv2 category. For 927 men with unilateral MR-visible lesions, SB CDR was additionally calculated for contralateral (SBc) and ipsilateral (SBi) subcohorts.

**Results:**

On CB, the CDR for csPCa was 54.8% (673/1229). CDR for csPCa was significantly higher for MRgFbx (50.0%, CI 47.1–52.8%) compared to SB (35.3%, CI 32.6–38.1%) for all PI-RADSv2 ≥ 3 categories (p < .05). The MRgFbx CDR for PI-RADSv2 categories 3, 4, and 5 were 81.5%, 88.5%, and 95.6% respectively. For unilateral lesion cases, significantly more csPCa was detected in the SBi compared to the SBc subcohort (30.1% (279/927) vs. 10.4%, (96/927), *p* < 0.001). The combination of MRgFbx and SBi detected csPCa in 97.0% (480) of the 495 csPCa detected by CB.

**Conclusion:**

MRgFbx had a higher CDR for csPCa than SB. While CB detected more csPCa than either method alone, in patients with a PI-RADSv2 category of 5, MRgFbx approximated the performance of CB. In unilateral lesion cases, SBc provided minimal added benefit.

## Introduction

Prostate cancer is the most common malignancy diagnosed in men worldwide [1]. Traditionally, prostate cancer has been diagnosed by a transrectal ultrasound guided systematic biopsy using 10–12 biopsy cores in a pre-defined template (SB). However, SB is associated with significant diagnostic inaccuracy: both under-diagnosis of significant prostate cancers and over-diagnosis of clinically insignificant prostate cancer [2, 3]. For patients who have a visible prostate lesion on multiparametric magnetic resonance imaging (mpMRI), MR guided fusion biopsy (MRgFbx) has been shown to have a higher cancer detection rate (CDR) than SB[4], likely due to the high sensitivity of Prostate Imaging-Reporting and Data System (PI-RADS) based scoring of MRI lesions [5–8]. While MRgFbx performs better than SB, it still misses significant prostate cancers. Use of combination biopsy (CB), incorporating SB and MRgFbx, has a higher CDR than either method alone [9, 10]. While CB has been established as the most robust biopsy method, it requires significantly more biopsy cores than standard SB or MRgFbx alone. Increasing biopsy core number is associated with increased risk of biopsy related adverse events including hematuria, hematospermia, perineal pain, and sepsis [11–13]. As such, there is a benefit to refining the protocols for prostate biopsy to include the minimum number of biopsy cores necessary for each patient to achieve a high detection rate. We analyzed a large single institution clinical cohort to explore potential approaches for reducing the number of biopsy cores while maintaining a high cancer detection rate. First, we explored the effect of Prostate Imaging-Reporting and Data System (PI-RADSv2) categories on the added value of SB. Second, we evaluated the added value of SB contralateral and ipsilateral to the MR target.

## Materials and methods

### Study design

This is an IRB approved and HIPAA compliant, single arm, observational study. Subjects were drawn consecutively from an integrated radiologic-pathologic report database of prostate biopsies performed for suspected prostate cancer between July 2016 and May 2020. Where men had multiple biopsies performed during this period, the first was included for analysis. Selection criteria included men with a positive 3TmpMRI without endorectal coil containing at least one lesion with PI-RADSv2 category of at least 3 who underwent prostate biopsy with SB of at least 10 cores and MRgFbx using the Artemis or Uronav systems. Exclusion criteria were lack of SB or incomplete SB (< 10 cores) or biopsy conducted with the InBore system. Combined biopsy (CB) was used as ground truth as prostatectomy specimens were not available.

A subset of patients with only unilateral targets were selected to analyze the laterality of positive biopsy cores in relation to MR targets. Any patients with midline lesions were excluded from this sub-analysis. SB was subdivided into contralateral (SBc) and ipsilateral (SBi) sub-cohorts.

### Imaging and biopsy methods

3TmpMRI was performed for all patients without endorectal coil one of several Siemens scanners (Siemens Healthineers, Erlangen, Germany): Prisma, Skyra, Trio, Vario with 40 channel body coils and high performance gradients. Technical specifications of the MRI comport with PI-RADSv2 recommendations. Interpretation was performed by sub-specialized genitourinary radiologists with significant experience in prostate MRI. Suspicious lesions were contoured using the ProFuse software (Eigen Inc) and graded using PI-RADSv2. MRI images and lesion contours were then loaded onto the fusion system.

The Artemis (Eigen, Grass Valley, CA) and UroNav (Philips Electronics, Amsterdam, The Netherlands) fusion biopsy systems were used. At the time of biopsy, a transrectal ultrasound was performed and the images were fused with the MR images. MRgFbx was performed by taking approximately 1 core for every 3 mm along the long axis of the target lesion. SB was obtained using a scalable grid. The targeting error at our institution has been previously shown to be up to 4 mm [14].

### Statistical analysis

The primary outcome was detection of clinically significant prostate cancer (csPCa), defined as Gleason Grade Group (GG) of 2 or higher. CDR were calculated for CB, MRgFbx and SB in the study cohort as well as sub-cohorts stratified by biopsy history [active surveillance (AS), biopsy naïve (BN), prior negative biopsy (PN)] and PI-RADSv2 category. Where not specified, the groups based on biopsy history and PI-RADSv2 category have been combined for analysis. For men with unilateral MR targets, SB CDR was subdivided into SBc and SBi sub-cohorts and CDR were calculated for each. 95% confidence intervals were computed by the Clopper-Pearson exact method and significance of difference between CDRs was assessed by McNemar’s test with continuity correction. Statistical analysis was performed in R and an alpha value of 0.05 was used for all significance tests.

## Results

### Patients

During the study period, 2439 men underwent prostate biopsy. Lesions with a PI-RADSv2 category > 2 were present for 1692 patients and 1378 ultimately underwent CB. The primary group of patients who underwent only MRgFbx without any systematic biopsy were those under active surveillance. Of patients who underwent CB, 1229 had a complete systematic biopsy of 10–12 cores. A sub-cohort of 927 men had only unilateral lesions. Among all the biopsies, an average of 11.7 SB cores were obtained, with 1–3 3TmpMRI targets/patient, and an average of 4.9 biopsy cores/MR target. Detailed patient characteristics are described in Table 1.

**Table 1:** Patient Imaging, Biopsy, and Lab characteristics.Performance of MR-targeted, systematic, and combined biopsy

Characteristic	Mean (SD); N
**Age**	65.7 (7.8)
**PSA free**	0.2 (1.2)
**PSA total**	4.9 (8.5)
**Biopsy history**	
Active surveillance	501
Biopsy naïve	623
Prior negative biopsy	105
**PI-RADSsv2 score**	
3	292
4	484
5	453
**Biopsy system**	
Artemis	934
UroNav	295

On CB, the CDR for csPCa was 54.8% (673/1229). CDR for csPCa was significantly higher for MRgFbx (50.0%, CI 47.1–52.8%) compared to SB (35.3%, CI 32.6–38.1%, p < 0.001). MRgFbx alone detected 614 (91.2%) of the 673 csPCa detected by CB, however the csPCa CDR for CB is significantly higher than MRgFbx (P < 0.001). For the 59 csPCa that would have been missed, 30 were classified as benign and 29 as non-significant prostate cancer (nsPCa), defined as GS of 6, on MRgFbx. For these missed cancer foci, the mean Gleason score was 7 and mean cancer core length (CCL) was 4.4 ± 3.1 mm. With the addition of SB, 63 biopsies which were characterized as benign on MRgFbx alone, were upgraded to nsPCa.

### Stratification by PI-RADSv2 categories

**Fig. 1:**
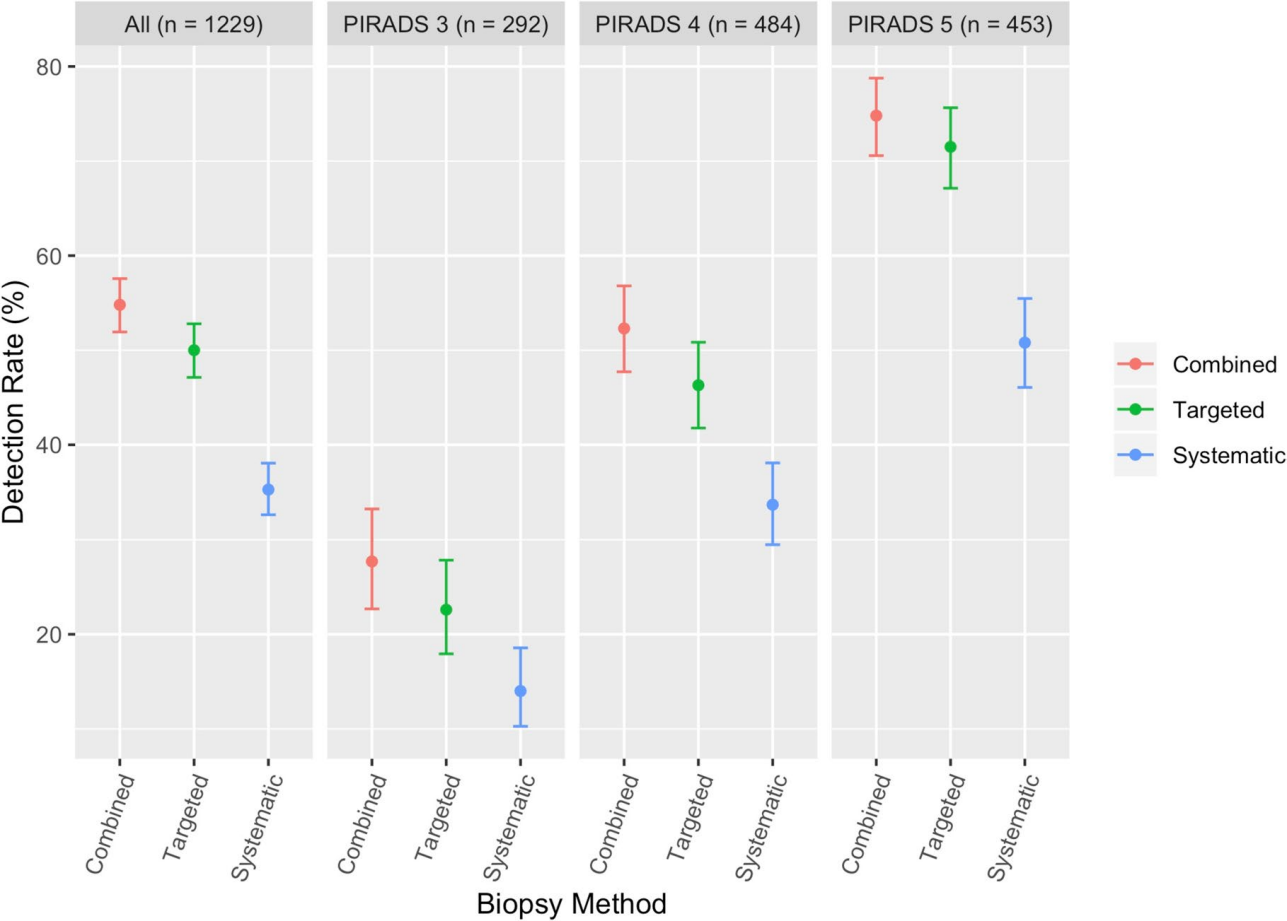
Cancer detection rate stratified by biopsy method and PI-RADSv2 category

For PI-RADS 3 lesions, the CB CDR for csPCa was 27.7% (81/292; Fig. 1). The CDR of csPCa was significantly higher for MRgFbx (22.6%, CI 17.9–27.8%) compared to SB (14.0%, CI, 10.3–18.6%, p < 0.005). MRgFbx alone detected 81.5% (66/81) of csPCa detected by CB, however the csPCa CDR for CB is significantly higher than MRgFbx (P < 0.001). For the 15 csPCa that would have been missed, 9 were classified as benign and 6 as nsPCa on MRgFbx and 27 of the biopsies which would have been characterized as benign on MRgFbx alone, were upgraded to nsPca with the addition of SB.

For PI-RADS 4 lesions, the CB CDR for csPCa was 52.3% (253/484). The CDR of csPCa was significantly higher for MRgFbx (46.3%, CI 41.8–50.8%) compared to SB (33.7%, CI 29.5–38.1%, p < 0.001). MRgFbx detected 88.5% (224/253) csPCa detected by CB, however the csPCa CDR for CB is significantly higher than MRgFbx (P < 0.001). For the 29 csPCa that would have been missed, 16 were classified as benign and 13 as nsPCa on MRgFbx. With the addition of SB, 27 biopsies which would have been characterized as benign on MRgFbx alone, were upgraded to nsPca.

For PI-RADS 5 lesions, the CB CDR for csPCa was 74.8% (339/453). The CDR of csPCa was significantly higher for MRgFbx (71.5%, CI 67.1–75.6%) compared to SB (50.8%, CI 46.1–55.5%, p < 0.001). MRgFbx detected 95.6% (324/339) csPCa detected by CB, however the csPCa CDR for CB is significantly higher than MRgFbx (P < 0.001). For the 15 csPCa that would have been missed, 5 were classified as benign and 10 as nsPCa on MRgFbx. With the addition of SB, nine biopsies which would have been characterized as benign on MRgFbx alone, were upgraded to nsPca.

### Stratification by biopsy history group

**Fig. 2:**
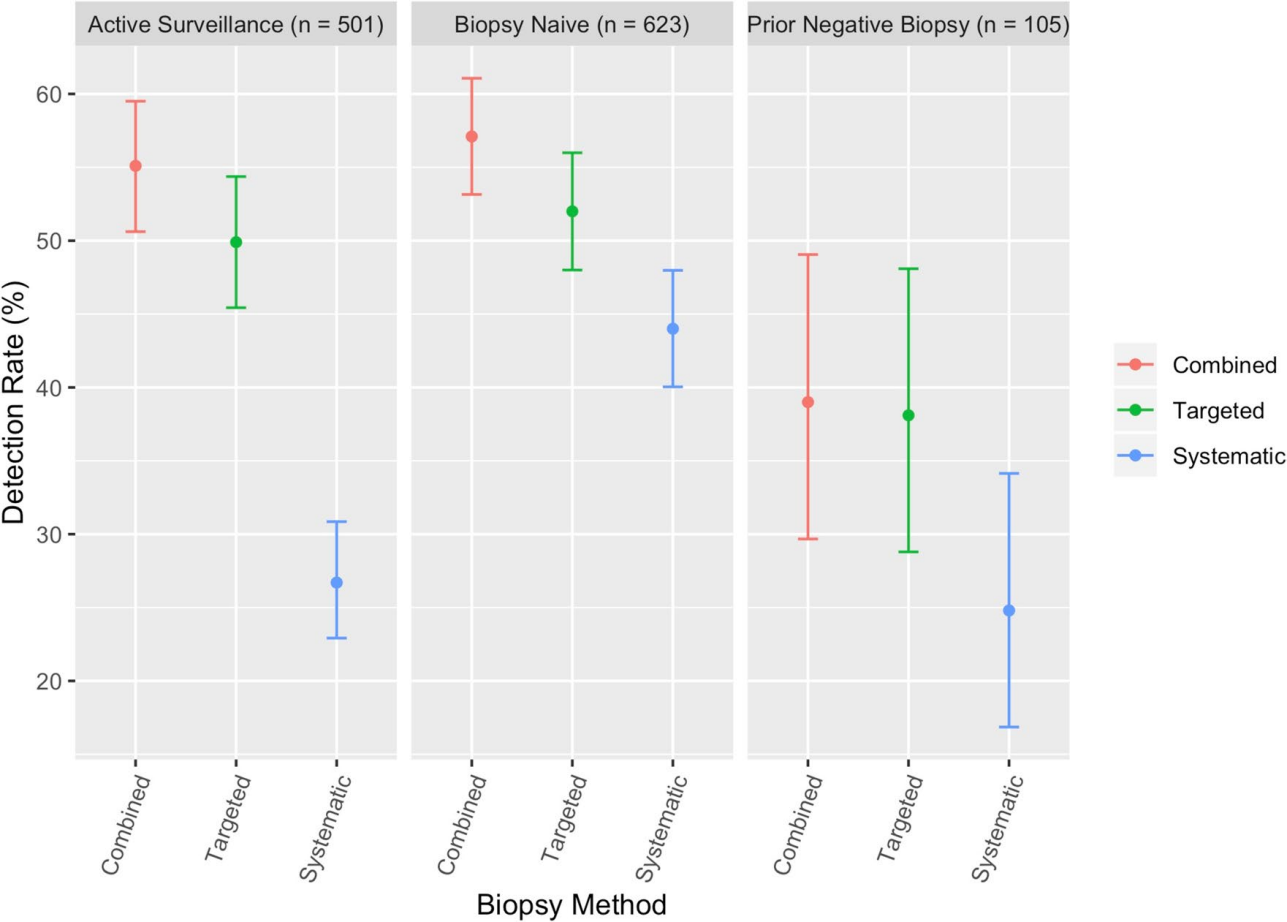
Cancer detection rate stratified by biopsy method and biopsy history group

For BN, the CB CDR for csPCa was 57.1% (356/623; Fig. 2). The CDR of csPCa was significantly higher for MRgFbx (52.0%, CI 48.0–56.0%) compared to SB (44.0%, CI 40.0–50.0%, p < 0.001). MRgFbx alone detected 91.0% (324/356) of csPCa detected by CB, however the csPCa CDR for CB is significantly higher than MRgFbx (p < 0.001). For the 32 csPCa that would have been missed, 22 were classified as benign and 21 as nsPCa on MRgFbx and 28 of the biopsies which would have been characterized as benign on MRgFbx alone, were upgraded to nsPca with the addition of SB.

For AS, the CB CDR for csPCa was 55.1% (276/501). The CDR of csPCa was significantly higher for MRgFbx (49.9%, CI 45.4–54.4%) compared to SB (26.7%, CI 22.9–30.9%, p < 0.001). MRgFbx detected 90.6% (250/276) of csPCa detected by CB, however the csPCa CDR for CB was significantly higher than MRgFbx (P < 0.001). For the 26 csPCa that would have been missed, 18 were classified as benign and 8 as nsPCa on MRgFbx. With the addition of SB, 28 biopsies which would have been characterized as benign on MRgFbx alone, were upgraded to nsPca.

For PN, the CB CDR for csPCa was 39.0% (41/105). The CDR of csPCa was significantly higher for MRgFbx (38.1%, CI 28.8–48.1%) compared to SB (24.8%, CI 16.9–34.1%, p < 0.005). MRgFbx detected 97.6% (40/41) csPCa detected by CB, however the csPCa CDR for CB is significantly higher than MRgFbx (P < 0.001). The one csPCa that would have been missed was classified as benign. With the addition of SB, seven biopsies which would have been characterized as benign on MRgFbx alone, were upgraded to nsPca.

**Fig. 3:**
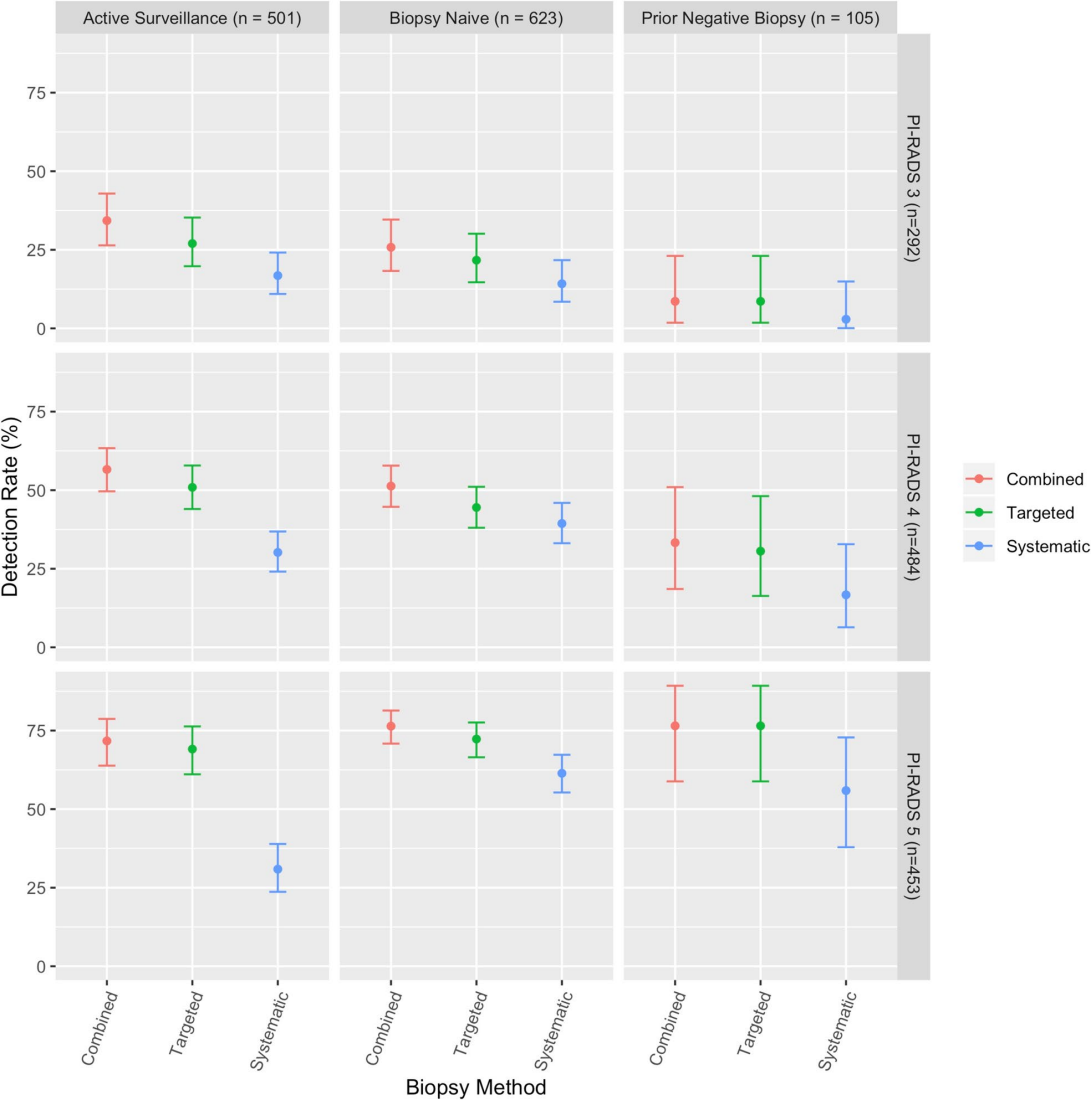
Cancer detection rate stratified by biopsy method, biopsy history group, and PI-RADSv2 category

A further post-hoc descriptive breakdown of CDR by both biopsy history and PI-RADS category is provided in Fig. 3. These results are provided as a granular description of the data.

### Added value of ipsilateral vs. contralateral systematic biopsy cores

**Fig. 4:**
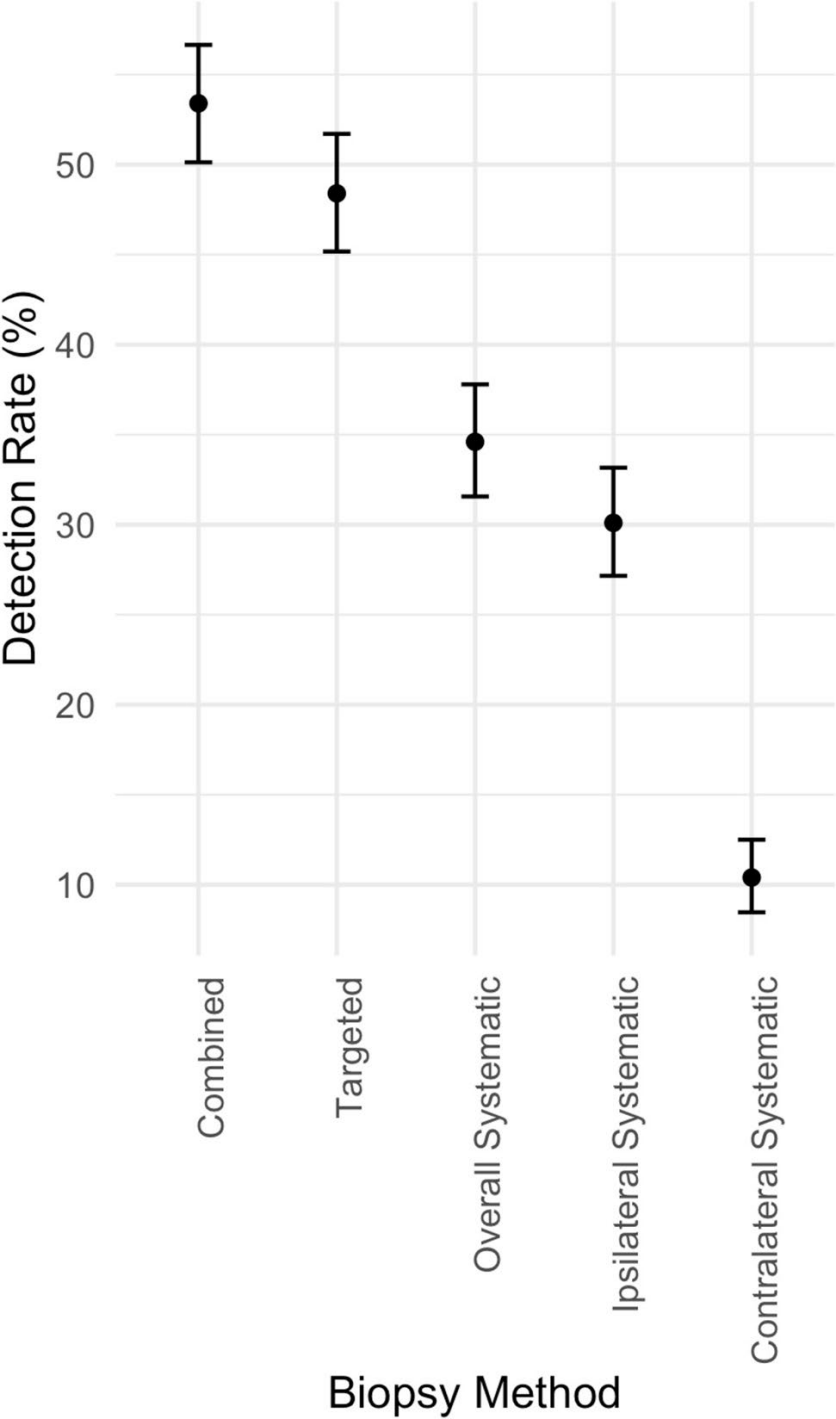
Cancer detection rate stratified by biopsy method, among patients with unilateral MRI visible lesions

In the CB cohort for men with unilateral MR targets, csPCa was detected in 53.4% (495/927) of biopsies. The MRgFbx subcohort CDR for csPCa was significantly higher than the SB subcohort alone (48.4% (449/927) vs 34.6% (327/927), *p* < 0.001). Significantly more csPCa was detected in the SBi compared to the SBc subcohort (30.1% (279/927) vs. 10.4%, (96/927), *p* < 0.001) (Fig. 4).

Of the csPCa detected on CB cohort, 9.3% (46/495) were missed by MRgFbx but detected by SB. Of these, 31, 13, and 2 were detected only by SBi, SBc, and both respectively. Overall, the combination of MRgFbx and SBi detected csPCa in 97.0% (480) of the 495 csPCa detected by CB. Pathological characteristics of missed cancer foci are described in Table 2. Further detailed characteristics of csPCa detected only by SBc are presented in Table 3. Of the csPCa in men with a PI-RADS 5 lesion, none were detected only by contralateral cores (Table 3). Performing MRgFbx with SBi alone would have saved an average of 5.91 ± 0.33 cores as compared to CB.

**Table 2:** Gleason score (GS) and cancer core length (CCL) of csPCa that were missed by MRgFbx but detected by SBi, SBc, or both

Biopsy method	n	GS	CCL (mm)
		Mean (SD)	Mean (SD)
SBi only	31	7.0 (.2)	5.0 (4.1)
SBc only	13	7.3 (.6)	3.4 (1.7)
Both	2	7 (0)	4.8 (1.8)

**Table 3:** Detailed characteristics of Cases Detected Only on Contralateral Cores, Gleason Group (GG), Cancer core length (CCL), Biopsy Naïve (BN), Active Surveillance (AS), Prior Negative Biopsy (PN)

# Contralateral cores with GG > 2	Gleason pattern; CCL (for highest GG core)	Biopsy history	PI-RADSv2
1	3 + 4; 7 mm (40%)	AS	3
1	3 + 4; 4 mm (50%)	AS	3
1	3 + 4; 5 mm (40%)	AS	3
1	3 + 4; 2 mm (20%)	AS	3
1	3 + 4; 2.5 mm (25%)	AS	3
3	4 + 5; 2.5 mm (20%)	BN	3
1	3 + 5; 2 mm (25%)	AS	4
1	4 + 3; 3 mm (30%)	AS	4
1	4 + 4; 2 mm (25%)	AS	4
2	3 + 4; 5 mm (30%)	BN	4
2	3 + 4; 1 mm (10%)	BN	4
1	4 + 3; 4.5 mm (75%)	BN	4
1	3 + 4; 4 mm, (40%)	PN	4

## Discussion

In this study we carefully analyzed a large clinical cohort using two widely utilized, commercially available fusion biopsy systems to identify the relationship of systematic biopsies to MR targets and determine whether an alternative to systematic biopsy could decrease the number of biopsy cores while achieving a high cancer detection rate. We found that in evaluating the effect of PI-RADSv2 category on the CDR of each biopsy method, for patients with a PI-RADSv2 category of 5 on 3TmpMRI, MRgFbx approximated the performance of CB with minimal added benefit for SB. Evaluating the CDR of each biopsy method in sub-cohorts divided by biopsy history found that for all groups MRgFbx performed significantly better than systematic biopsy, with the most pronounced effect among men under active surveillance. The likelihood and location of occurrence for csPCa can differ depending on clinical history and this finding improves the applicability of our results to all men undergoing biopsy. We next analyzed where in the prostate SB cores are most beneficial, finding that among patients with unilateral lesions only, the combination of MRgFbx and a wider ipsilateral systematic biopsy detected 97% of csPCa detected on CB. Furthermore, for unilateral lesions with a PI-RADSv2 suspicion category of 5, all csPCa was detected by the combination of MRgFbx with ipsilateral systematic biopsy. Each of these approaches suggest that high csPCa detection rates may be obtained with less overall sampling if, in PI-RADSv2 category 5 cases, systematic sampling is omitted, and if in unilateral lesion cases, contralateral systematic sampling is omitted.

As a baseline comparison of overall cancer detection rates for each of the three biopsy methods, we found that in contrast to the MRI-FIRST trial [9], but consistent with the PRECISION trial [4] and work by Siddiqui et al. [15], the cancer detection rate for targeted biopsy in this study was significantly higher (91.2%) than that of systematic biopsy (64.5%) (Table 4). The cancer detection rate for combined biopsy, 54.8%, is higher in our sample than in prior studies, e.g. 43.6% for Ahdoot et al. [16] and 37.5% in the MRI-FIRST trial [9]. This difference may result from differences in patient cohorts, since our study includes repeat biopsy patients for confirmation or active surveillance rather than biopsy naïve patients only. In addition, the use of the Artemis system for the majority of study subjects, which provides a spatially optimized map for systematic biopsy may also contribute to higher rates of cancer detection.

**Table 4:** Summary of three major studies evaluating systematic and targeted prostate biopsies

Study	PRECISION	MRI-FIRST	Ahdoot, et. al., NEJM (2020)
n	500	251	2103
Systematic Biopsy Method	10–12 cores	12 cores plus up to 2 cores for hypoechoic US targets	
Targeted Biopsy Method	Maximum 3 targets with up to 4 cores per target; visual targeting or MR-US fusion software	Maximum 2 targets; 3 cores per target; cognitive guidance or MR-US fusion software	
Systematic Biopsy CDR (csPCa*)	26%	29.9%	31.0%
Systematic Biopsy CDR (nsPCa)	22%	19.5%	21.6%
Targeted Biopsy CDR (csPCa)	38%	32.3%	37.8%
Targeted Biopsy CDR (nsPCa)	9%	5.6%	13.7%
Combined Biopsy CDR (csPCa)	NA	37.5%	43.6%

^*^Definitions of clinically significant prostate cancer differ across the studies but the detection rates shown here are reported for Gleason score 7 or higher prostate cancer

The current report is a more lesion-level analysis than prior studies from this institution, including Elkhoury et al. and Sonn et al [17, 18]. Contrasting the current findings, the PAIREDCAP trial concluded that targeted and systematic biopsies have similar CDR [17]. Key differences in the studies may explain discrepant conclusions. The PAIREDCAP sample included only biopsy naïve patients, whereas the current sample includes patients on active surveillance and with prior negative biopsies. Biopsies in the PAIREDCAP trial were performed by one experienced operator and included both cognitive and software-based fusion approaches. Biopsies in this report were performed by a variety of operators using the Artemis and UroNav systems. The difference in biopsy approach and possible influence of procedural learning curve may explain additional discrepancy.

The findings that MRgFbx detected up to 95.6% of total csPCa for PI-RADSv2 category 5 lesions agrees with prior studies showing that CDR for MRgFbx increases with imaging suspicion [6, 17, 18]. The MRI-FIRST trial reported CDR for systematic biopsy, targeted biopsy, and combined biopsy stratified by level of imaging suspicion using an institutional Likert scale [9]. Their results showed that for a Likert score of 4 out of 5, 22 of the 23 (95.7%) instances of csPCa captured on CB were found by MRgFbx alone and for a score of 5, 51 of 55 (92.7%) csPCa on CB were detected by MRgFbx alone. The MRI-FIRST trial utilized a smaller sample size than this study and used both cognitive fusion and software-based fusion for targeted biopsy. Despite these differences, our data bolsters the finding that for high levels of imaging suspicion, targeted biopsy approaches the performance of combined biopsy. This study highlights that PI-RADSv2 scoring, when applied to a large cohort, demonstrates similar, if not greater sensitivity to underlying cancer rates than institutional Likert scores.

Our data additionally shows that for men with unilateral lesions on MRI, omitting contralateral SB would reduce the CDR by only 3% compared to CB and have no benefit for PI-RADS category 5 lesions, while using an average of 5.91 fewer cores. Few studies to date have closely examined the added benefit of contralateral sampling in unilateral lesion cases with stratification by PI-RADSv2 categories in a large cohort. A series of smaller cohort studies have shown high CDRs for targeted biopsy (particularly for high suspicion PI-RADS categories) and minimal added benefit of contralateral cores [19–24]. Specifically evaluating patients with a PIRADS 5 lesion, a study of 119 patients recommends avoiding contralateral lobe systematic biopsy, since in this subset of cases they observed no loss of prostate cancer diagnoses with the exclusion of contralateral cores [24]. One larger report of 753 patients (572 with a solitary lesion) also concludes that targeted and ipsilateral biopsy is a promising biopsy schema, however lesions that cross midline and multifocal lesions without laterality specification for the second lesion are included in one overall analysis [19]. In contrast to the above detailed studies, reports have also shown csPca detected by contralateral SB in a relatively high 8.5% and 7.3% of 130 and 212 patient cohorts, respectively [25, 26]. Based on these studies, further clarification of the role of ipsilateral and contralateral biopsy cores in a large cohort is needed.

The combination of MRgFbx and ipsilateral systematic cores is one version of a regional targeted biopsy, a promising approach with growing interest in the literature. Raman et al. (2021) proposed a biopsy approach using cores within 2 cm of the margin of the MRI target. In a study of 971 men, this approach did not significantly differ from MRgFbx but used 3.8 fewer cores on average [27]. A recent study by Brisbane et al. (2022) shows that 90% of csPCa cores are captured within a 10 mm penumbra around the MRI target and that with increasing PI-RADS category, the size of the penumbra required to capture 90% of csPCa decreases (e.g. requiring only 5 mm distance around a PI-RADS 5 lesion) [28]. By considering the ipsilateral cores in the present study as a penumbra around the target, and observing where cancer negative cores occur, this could delineate an appropriate zone for focal therapy. The exclusion of patients with midline lesions on MRI, as performed in this study, is an important consideration, since doing so has been shown to significantly decrease the possibility of patients in the study group having contralateral lesions [29]. Determining the presence of contralateral lesions is an important factor in assessing eligibility for focal therapies [30]. This study supports the addition of ipsilateral SB alone to MRgFbx for csPCa diagnosis and in evaluation for hemi-gland ablations for patients with unilateral lesions.

## Limitations

Our study has multiple limitations. First, due to the retrospective nature of this analytic approach, we cannot prospectively assess the alternate biopsy protocols proposed. Next, since the patient cohort in this study consisted of both biopsy-naïve men as well as men on active surveillance, and the pretest probability of cancer occurrence differs between these groups, this may limit the generalizability of results from combined analyses when looking solely at biopsy naïve men or solely at men on active surveillance. In addition, the use of PI-RADSv2 instead of PI-RADSv2.1 in this study could affect cancer detection rates between PI-RADS categories 3–5 and this study also does not include a sub-analysis based on patients with 2 or more targets. No correction for multiple testing was performed. Lastly, the biopsies included in this study were all performed at a single academic institution with expertise in prostate MR, pathology, and biopsy, which may not be broadly available.

## Conclusion

The optimal number and location of prostate biopsy cores remains controversial as increasing the number of cores may cause added morbidity, with growing interest in the literature in approaches such as the ‘regional targeted biopsy’ which use MRI delineated lesions to focus the biopsy approach. In this paper, we propose two strategies based on a large clinical dataset for reducing the number of cores necessary to maintain a high CDR: 1) limiting biopsies to MRgFbx for patients with high levels of imaging suspicion based on PI-RADSv2 and 2) using MRgFbx plus an ipsilateral SB without the contralateral cores for patients with definitively unilateral lesions on MRI, which represents one version of a regional targeted biopsy approach. These results suggest that the two proposed alternate biopsy protocols may be useful for reducing morbidity in prostate biopsy and merit additional study for prospective assessment.

## Data Availability

Data may be publically available via the UCLA Integrated Diagnostics Program after formal application and request to corresponding author.
